# The complete mitochondrial genome of *Sarcophaga scopariiformis* (Diptera: Sarcophagidae)

**DOI:** 10.1080/23802359.2020.1787269

**Published:** 2020-07-10

**Authors:** Weifeng Wang, Chaojun Wang, Yueting She, Qin Zeng, Wei Mao, Xiaoping Gu, Hao Yuan, Xiaoling Pan, Yong Wang

**Affiliations:** a The Key Laboratory of Model animals and Stem Cell Biology in Hunan Province (2019TP1035), School of Medicine, Hunan Normal University, Changsha, PR China; b Department of Forensic Science, School of Basic Medical Science, Central South University, Changsha, PR China

**Keywords:** Sarcophagidae, *Sarcophaga scopariiformis*, mitochondrial genome

## Abstract

*Sarcophaga scopariiformis* (Diptera: Sarcophagidae), a potential vector of pathogens as well as one of the important flesh fly specie in forensic entomology. We have firstly sequenced and assembled the whole mitogenome of *S. scopariiformis* in this study. The circular mitogenome is 15,325 bp in length, consisting of A (39.3%), G (9.4%), T (36.8%), and C (14.5%). It showed in a typical mitochondrial genome similar to other sarcophagids species, which is composed of 13 protein-coding genes (PCGs), 22 transfer *RNA* genes (tRNA), 2 ribosomal *RNA* genes (rRNA), and a non-coding AT-rich region. Moreover, the phylogenetic analysis based on 13 PCGs has been conducted. The topological structure of the phylogenetic tree clearly indicated that *S. scopariiformis* was more closed to the species of *Sarcophaga peregrina* in genetic distance, compared to the other sarcophagids species. This study provided a useful data reference for genetic structure and phylogenetic analysis of Sarcophagidae.

As a member of Sarcophagidae family and Diptera order (Thompson and Pape 2016), *Sarcophaga scopariiformis* Senior-White, 1927, mainly presents in geographic regions of Asia such as China, Thailand, Laos, and Sri Lanka. The adults are potential vectors of pathogens (Greenberg 1971), which is important in medical and sanitary science. While the larvae feed on dead organic matter, such as the carrion, feces, and decaying substances. Thus, the background information of growth and developing stage of *S. scopariiformis* could facilitate forensic pathologist to estimate the minimum postmortem interval (PMImin) if their eggs or larvae are found on the carcass. The correct identification of insect species is an essential step of forensic entomology since the closely related sarcophagids species may differ substantially in growth rate and diapauses responses. Molecular species identification method greatly improved the identification efficiency for forensic pathologists. The mitochondrial genome (mitogenome) has been widely used as a genetic marker in species identification, evolutionary biology, and molecular ecology.

In this study, the specimens of *S. scopariiformis* were collected at Changsha city, Hunan province, PR China (the geospatial coordinates: 28.25°N, 112.55°E) on 20 November 2019. The specimen was preserved in the laboratory at −80 °C (Changsha, Hunan, China) with a sole code (CSU2020050790). Specimen identification was conducted according to traditional morphological approaches and then total genomic DNA was extracted from a single specimen, with the QIANamp Micro DNA Kit according to the manufacturer’s protocol, and the subsequent high-throughput genome sequencing was conducted on an Illumina HiSeq 2500 platform, with a strategy of 150 paired-end sequencing. We employed MITObim version 1.9 to perform the *de novo* assembly and annotation (Hahn et al. 2013; Ren et al. 2019).

The newly determined mitogenome sequence of *S. scopariiformis* has been submitted to GenBank under the accession number MT476486. The circular mitogenome is 15,325 bp in length, consisting of A (39.3%), G (9.4%), T (36.8%), and C (14.5%). It showed in a typical mitochondrial genome similar to other sarcophagids species, which is composed of 13 protein-coding genes (PCGs), 22 transfer *RNA* genes (tRNA), 2 ribosomal *RNA* genes (rRNA), and a non-coding AT-rich region.

Phylogenetic analyses were conducted using neighbor-joining (NJ) tree inference method *via* software of MEGA version 7.0 (Kumar et al. 2016), based on 13 PCGs of the newly determined *S. scopariiformis* and 11 published *Sarcophaga* species mitogenomes, in which the sequence of *Calliphora vomitoria* and *Chrysomya pinguis* were set as the outgroups (Figure 1). The topological structure of the phylogenetic tree clearly indicated that *S. scopariiformis* was more closed to the species of *Sarcophaga peregrina* in genetic distance, compared to the other sarcophagids species. Our study reported the mitogenome of *S. scopariiformis*, which provided a useful data reference for genetic structure and phylogenetic analysis of Sarcophagidae and the better understanding of the genetic relationship of flesh flies.

**Figure 1.**
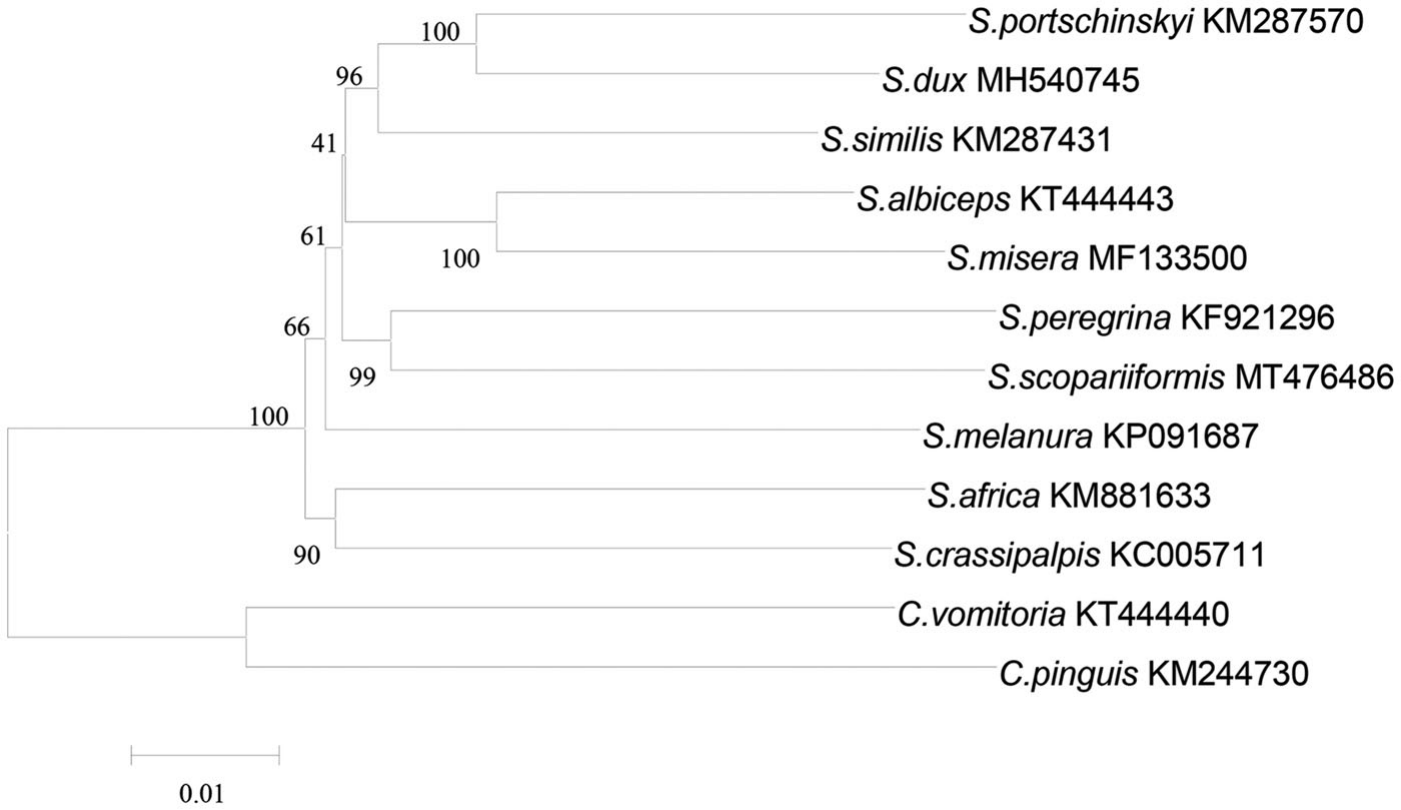
Phylogenetic analyses of 12 sarcophagids species were constructed using NJ method based on 13 PCGs. Morphological species identification and voucher ID were given in the label. Numbers on branches showed the bootstrap support value. The out-group consists of two specimens of *Calliphora vomitoria* and *Chrysomya pinguis*.

## Data Availability

The data that support the findings of this study are openly available in GenBank at https://www.ncbi.nlm.nih.gov, reference number MT476486.
